# Functional and clinical outcome after operative versus nonoperative treatment of a humeral shaft fracture (HUMMER): results of a multicenter prospective cohort study

**DOI:** 10.1007/s00068-022-01890-6

**Published:** 2022-02-09

**Authors:** Dennis Den Hartog, Saskia H. Van Bergen, Kiran C. Mahabier, Michael H. J. Verhofstad, Esther M. M. Van Lieshout, the HUMMER Investigators, Ivo Beetz, Hugo W. Bolhuis, P. Koen Bos, Maarten W. G. A. Bronkhorst, Milko M. M. Bruijninckx, Jeroen De Haan, Axel R. Deenik, P. Ted Den Hoed, Martin G. Eversdijk, J. Carel Goslings, Robert Haverlag, Martin J. Heetveld, Albertus J. H. Kerver, Karel A. Kolkman, Peter A. Leenhouts, Sven A. G. Meylaerts, Ron Onstenk, Martijn Poeze, Rudolf W. Poolman, Bas J. Punt, Ewan D. Ritchie, W. Herbert Roerdink, Gert R. Roukema, Jan Bernard Sintenie, Nicolaj M. R. Soesman, Edgar J. T. Ten Holder, Wim E. Tuinebreijer, Maarten Van der Elst, Frank H. W. M. Van der Heijden, Frits M. Van der Linden, Peer Van der Zwaal, Jan P. Van Dijk, Hans-Peter W. Van Jonbergen, Egbert J. M. M. Verleisdonk, Jos P. A. M. Vroemen, Marco Waleboer, Philippe Wittich, Wietse P. Zuidema, Ahmed Al Khanim, Jelle E. Bousema, Kevin Cheng, Yordy Claes, J. Daniël Cnossen, Emmelie N. Dekker, Aron J. M. De Zwart, Priscilla A. Jawahier, Boudijn S. H. Joling, Cornelia A. W. Notenboom, Jaap B. Schulte, Nina Theyskens, Gijs J. J. Van Aert, Boyd C. P. Van der Schaaf, Tim Van der Torre, Joyce Van Veldhuizen, Lois M. M. Verhagen, Maarten Verwer, Joris Vollbrandt

**Affiliations:** Trauma Research Unit, Department of Surgery, Erasmus MC, University Medical Center Rotterdam, P.O. Box 2040, 3000 CA Rotterdam, The Netherlands

**Keywords:** Fracture, Humerus, Nonoperative, Outcome, Shaft, Treatment

## Abstract

**Purpose:**

The best treatment of humeral shaft fractures in adults is still under debate. This study aimed to compare functional and clinical outcome of operative versus nonoperative treatment in adult patients with a humeral shaft fracture. We hypothesized that operative treatment would result in earlier functional recovery.

**Methods:**

From October 23, 2012 to October 03, 2018, adults with a humeral shaft fracture AO type 12A or 12B were enrolled in a prospective cohort study in 29 hospitals. Patients were treated operatively or nonoperatively. Outcome measures were the Disabilities of the Arm, Shoulder, and Hand score (DASH; primary outcome), Constant–Murley score, pain (Visual Analog Score, VAS), health-related quality of life (Short Form-36 (SF-36) and EuroQoL-5D-3L (EQ-5D)), activity resumption (Numeric Rating Scale, NRS), range of motion (ROM) of the shoulder and elbow joint, radiologic healing, and complications. Patients were followed for one year. Repeated measure analysis was done with correction for age, gender, and fracture type.

**Results:**

Of the 390 included patients, 245 underwent osteosynthesis and 145 were primarily treated nonoperatively. Patients in the operative group were younger (median 53 versus 62 years; *p* < 0.001) and less frequently female (54.3% versus 64.8%; *p* = 0.044). Superior results in favor of the operative group were noted until six months follow-up for the DASH, Constant–Murley, abduction, anteflexion, and external rotation of the shoulder, and flexion and extension of the elbow. The EQ-US, and pronation and supination showed superior results for the operative group until six weeks follow-up. Malalignment occurred only in the nonoperative group (*N* = 14; 9.7%). In 19 patients with implant-related complications (*N* = 26; 10.6%) the implant was exchanged or removed. Nonunion occurred more often in the nonoperative group (26.3% versus 10.10% in the operative group; *p* < 0.001).

**Conclusion:**

Primary osteosynthesis of a humeral shaft fracture (AO type 12A and 12B) in adults is safe and superior to nonoperative treatment, and should therefore be the treatment of choice. It is associated with a more than twofold reduced risk of nonunion, earlier functional recovery and a better range of motion of the shoulder and elbow joint than nonoperative treatment. Even after including the implant-related complications, the overall rate of complications as well as secondary surgical interventions was highest in the nonoperative group.

**Trial registration:**

NTR3617 (registration date 18-SEP-2012).

**Supplementary Information:**

The online version contains supplementary material available at 10.1007/s00068-022-01890-6.

## Background

Humeral shaft fractures account for 1–3% of all fractures [1]. The incidence rate is 14.5 per 100,000 persons per year with a gradually increasing age-specific incidence from the fifth decade, reaching almost 60/100,000 per year in the ninth decade [1].

Last decade, the optimal treatment for humeral shaft fractures was subject to debate. A recent meta-analysis shows that satisfactory results can be achieved with both nonoperative and operative management [2]. The meta-analysis of data from randomized controlled trials (RCTs) in their review showed no statistically significant differences in favor of either one of the treatment options. Operative and nonoperative treatment each have their individual advantages and disadvantages. Surgical treatment is mostly performed using intramedullary nailing or plating, and the mostly used nonoperative treatment is immobilization with a functional (Sarmiento) brace or a cast [3]. Fracture fixation allows for early mobilization, and is aimed to achieve earlier functional recovery. A disadvantage is the risk of surgical complications [4]. Nonoperative treatment is aimed to achieve secondary bone healing by temporary immobilization of the arm. This initially results in functional impairment and may delay functional recovery. Moreover, the indirect fracture stabilization and risk of inadequate fracture alignment may increase the risk of malunion and nonunion [5, 6]. Nonunion occurs in up to 10% of patients treated operatively and in up to 23% of patients treated nonoperatively [2, 5, 6]. A complication that may occur after a humeral shaft fracture is radial nerve palsy. A systematic review reported an average radial nerve palsy rate at presentation of 11.8% in 4517 patients [7]. The reported rate of radial nerve palsy due to surgery was 3.5% [2]**.**

The finding that the rate of surgical treatment was approximately 50% across all AO fracture subtypes indicates that consensus on the best treatment strategy for humeral shaft fractures was lacking at the time the study was designed [8]. Lack of confirmative evidence about the best treatment strategy was also concluded in a Cochrane review [9]. A survey among members of the British Elbow and Shoulder Society in 2021 concluded that the management preference for humeral shaft fractures among surgeons is highly variable, and that this may be partly attributed to the sparsity of high-quality evidence. They proposed that well-designed prospective cohort studies or randomized trials may guide further management of these injuries [10]. The current study was designed to provide such high-quality evidence. We hypothesized that operative treatment would result in earlier functional recovery.

The primary objective of this study was to examine the effect of operative versus nonoperative treatment on the Disabilities of the Arm, Shoulder, and Hand (DASH) score, reflecting functional outcome and pain of the upper extremity, in adult patients who sustained a humeral shaft fracture. Secondary aims were to examine the effect of treatment on functional outcome (Constant–Murley) score, level of pain, range of motion of the shoulder and elbow joint, occurrence of complications with associated interventions, health-related quality of life, and the time to resumption of work and activities of daily living in these patients.

## Methods

### Setting and participants

The HUMMER study was a multicenter, parallel group cohort study, conducted in 29 hospitals in The Netherlands. All persons aged 18 years or older presenting to the Emergency Department (ED) with a humeral shaft fracture (AO type 12A or 12B on plain radiographs) were eligible for inclusion [11]. Primary osteosynthesis had to be performed within 14 days after presentation to the ED. Patients were excluded if they had (1) concomitant injuries affecting treatment and rehabilitation of the affected arm; (2) a humeral fracture treated with an external fixator; (3) a pathological, recurrent, or open humeral shaft fracture; (4) neurovascular injuries requiring immediate surgery (excluding radial nerve palsy); (5) additional traumatic injuries of the affected arm that would influence upper extremity function; (6) an impaired upper extremity function prior to the injury; (7) retained hardware around the affected humerus; (8) rheumatoid arthritis; or (9) a bone disorder which would impair bone healing (excluding osteoporosis). Patients with expected problems in maintaining follow-up or with insufficient Dutch language proficiency were also excluded. Exclusion of a patient because of enrollment in another drug or surgical intervention trial was left to the discretion of the attending surgeon on a case-by-case basis. The study was exempted by the Medical Research Ethics Committees and Local Ethics Boards of all participating centers. The study protocol is available online [12].

### Treatment allocation and masking

Eligible patients were informed about the study after presentation to the ED and could be enrolled until their first outpatient department visit 14 days after trauma. Patients were treated operatively or nonoperatively, as per decision of the patient and treating surgeon. All surgeons were certified (orthopedic) trauma surgeons with extensive experience in fracture care. Plaster casts or braces were applied by experienced orthopedic or plaster technicians.

Masking participants or investigators for treatment was not possible. To reduce bias, the follow-up measurements were standardized. Radiographs were evaluated independently by two assessors (IB and DDH). In case of disagreement, consensus was reached after discussion.

### Intervention

If a surgeon decided to perform osteosynthesis, the approach for fracture reduction (open or closed), fixation (antegrade or retrograde nailing, or open or minimally invasive plate osteosynthesis), the type and brand of the materials as well as the use of cerclage wires and other add-ons were left to the surgeon. Critical elements of this treatment (*e.g.*, type of implant, surgical approach, operative delay, and duration of surgery) were recorded.

The type of nonoperative treatment was also left to the attending surgeon. Usually it consisted of a splint, collar and cuff or (hanging) cast for 1–2 weeks, followed by a Sarmiento brace for 4–6 weeks. Critical elements of this treatment were also recorded.

Due to a lack of evidence favoring a particular approach, the physical therapy and rehabilitation program was recorded but not standardized.

### Assessments and follow-up

Follow-up data were obtained during outpatient visits at two weeks (7–21 days window), six weeks (4–8 weeks window), three months (11–15 weeks window), six months (6–7 months window), and 12 months (12–14 months window) after start of treatment. At each visit, the investigators recorded clinical data from the patient files (*e.g.*, complications and treatment) and measured the range of motion of the shoulder and elbow. At each visit, patients were asked to complete a set questionnaires on their level of pain, functional recovery, activity resumption, and health-related quality of life (HR-QoL). From six weeks onwards, the investigators determined the Constant–Murley score. As part of routine care, anterior–posterior and lateral radiographs of the humerus were made at the time of hospital presentation, after reduction, and at each subsequent hospital visit.

The primary outcome measure was the Disabilities of the Arm, Shoulder and Hand (DASH) score [13, 14]. Secondary outcome measures were the Constant–Murley score [15], level of pain (Visual Analog Scale, VAS), analgesic drugs used, Range of Motion (ROM) of the shoulder and elbow joint, time to resumption of work, resumption of activities of daily living (Numeric Rating Scale, NRS), health-related quality of life (Short Form-36 Physical Component Summary (SF-36 PCS) and Mental Component Summary (SF-36 MCS), and EuroQoL-5D-L Utility Score (EQ-5D US) and Visual Analog Scale (EQ-5D VAS)) [16–18], the occurrence of complications with associated secondary interventions, and radiologic healing. Nonunion is defined as a failure to heal at 26 weeks post fracture with no progress toward healing seen on the most recent radiographs [19]. This was determined from radiographs by two experienced trauma surgeons independently. ROM was measured by trained research physicians or research assistants using a goniometer and a standardized protocol. The patient-reported outcome measures were all available in Dutch and were proven reliable, valid, and responsive in the studied population [20, 21]. A detailed description of these questionnaires can be found in the published study protocol [12].

At baseline, patient characteristics, such as age, gender, American Society of Anesthesiologists' (ASA) classification, smoking, comorbidities, dominant side, medication use, and work and sports participation pre-trauma, were collected. Also, injury-related variables (such as the affected side, mechanism of injury, and fracture classification (according to the AO classification system) [11], and additional injuries) were recorded.

### Statistical analysis

Sample size calculation for the primary analysis was based on the assumption that the mean DASH in the nonoperative group would be 16, with a Standard Deviation (SD) of 16 [22]. We expected a DASH score of 10 (SD 10) in the operative group at three months [22]. A two-sided test with an α level of 0.05 and a β level of 0.2 required 78 patients in both treatment groups. To account for loss of patients due to mortality (10%) and loss to follow-up (10% anticipated based upon previous studies by the research team), a sample size of 95 patients per group would suffice. To allow for subgroup analysis for the most common AO fracture subtypes, 400 patients were targeted. This was based on the relative occurrences of the AO fracture subtypes as found in a retrospective study [8].

Analyses were performed using the Statistical Package for the Social Sciences (SPSS) version 25. Analysis was by intention to treat and all statistical tests were two-sided. The study is registered at the Netherlands Trial Register (NTR3617). Missing data were not imputed. Normality of continuous data was assessed using the Shapiro–Wilk test, and homogeneity of variances across groups was tested with the Levene’s test. Chi-squared analysis was used for statistical testing of categorical data. Continuous data were analyzed using a Mann–Whitney *U *test. *P *values < 0.05 were regarded as statistically significant.

Continuous outcomes that were repeatedly measured over time were compared between treatment groups using linear mixed-effects regression models. These multilevel models included random effects for the intercepts of the model and time coefficient of individual patients. Since the outcome measures were not linearly related with time, the time points were entered as factor. The models included fixed effects for treatment group, age, gender, and the individual fracture types. The effect of a fracture at the dominant side, smoking, and radial nerve palsy at trauma was non-significant in all models and these covariates were therefore not included. As most participating hospitals used both treatment strategies, study site was also not included in the model. The interaction between treatment group and time was included in the model to test for differences between the groups over time. For each follow-up moment, the estimated marginal mean was computed per treatment group and compared post hoc using a Bonferroni test to correct for multiple testing. Absence of overlap in the 95% confidence interval around the marginal means was regarded as significant at *p* < 0.05.

## Results

### Patient and injury characteristics

**Fig. 1:**
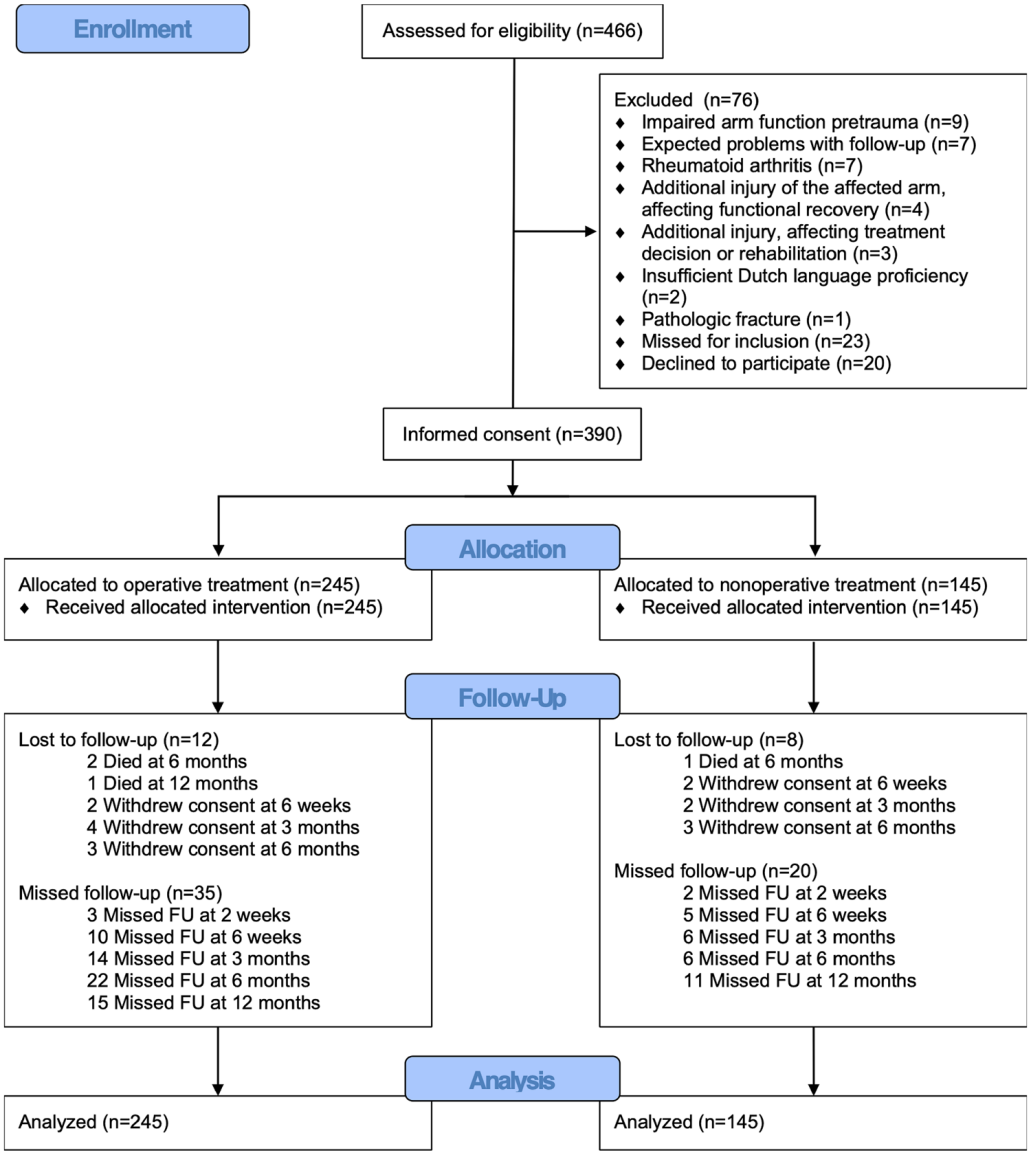
Flow chart of the study

Between October 23, 2012 and October 03, 2018, 466 patients were screened for eligibility, of whom 390 were included. Main exclusion reasons were an impaired arm function before trauma (*N* = 9), expected problems with follow-up (*N* = 7), and rheumatoid arthritis (*N* = 7). Twenty patients declined to participate, and 23 were screened too late and were thus recorded as missed. Of the included patients, 245 were operated and 145 underwent nonoperative treatment (Fig. 1). All patients received the allocated treatment. Twenty patients were lost to follow-up due to mortality (*N* = 4) or withdrawal of consent (nine in the operative group and seven in the nonoperative group). Thirty-five in the operative group and 20 patients in the nonoperative group did not show up at least one follow-up visit (Fig. 1).

The two treatment groups had similar baseline and injury characteristics, except for a relative underrepresentation of females (*N* = 133 (54.3%) versus *N* = 94 (64.8%); *p* = 0.044) and patients with osteoporosis or osteopenia (*N* = 1 (0.4%) versus *N* = 5 (3.4%); *p* = 0.028), and a lower median age (53 (*P*_25_–*P*_75_ 35–66) versus 62 (*P*_25_–*P*_75_ 49–71) years; *p* < 0.001) in the operative group (Table 1). Fractures in the operative group were less often A1 (*N* = 57 (23.3%) versus *N* = 51 (35.2%)) or B1 (*N* = 51 (20.8%) versus *N* = 42 (29.0%)), and more often A3 (*N* = 71 (29.0%) versus *N* = 18 (12.4%); *p* = 0.002).

**Table 1:** Patient, injury, treatment, and admission details of study participants by treatment group

	N*	All (*N* = 390)	N*	Operative (*N* = 245)	N*	Nonoperative (*N* = 145)	*P* value
Patient characteristics							
Female	390	227 (58.2%)	245	133 (54.3%)	145	94 (64.8%)	**0.044**
Age (year)	390	57 (40–68)	245	53 (35–66)	145	62 (49–71)	** < 0.001**
BMI (kg/m^2^)	387	26.0 (23.4–29.7)	244	26.1 (23.4–29.9)	143	25.8 (23.4–29.6)	0.928
Smoking	390	81 (20.8%)	245	55 (22.4%)	145	26 (17.9%)	0.304
ASA 3 or 4	390	25 (6.4%)	245	13 (5.3%)	145	12 (8.3%)	0.286
Comorbidities							
Any	390	198 (50.8%)	245	115 (46.9%)	145	83 (57.2%)	0.059
Diabetes	390	30 (7.7%)	245	18 (7.3%)	145	12 (8.3%)	0.844
Osteoarthritis	390	32 (8.2%)	245	15 (6.1%)	145	17 (11.7%)	0.058
Osteoporosis or osteopenia	390	6 (1.5%)	245	1 (0.4%)	145	5 (3.4%)	**0.028**
Medication use	390	208 (53.3%)	245	127 (51.8%)	145	81 (55.9%)	0.463
Number of medications	208	3 (1–5)	127	2 (1–4)	81	3 (1–5)	0.225
Injury characteristics							
Dominant side affected	390	189 (48.5%)	245	116 (47.3%)	145	73 (50.3%)	0.601
Fracture classification							
A1	390	108 (27.7%)	245	57 (23.3%)	145	51 (35.2%)	**0.002**
A2		66 (16.9%)		43 (17.6%)		23 (15.9%)	
A3		89 (22.8%)		71 (29.0%)		18 (12.4%)	
B1		93 (23.8%)		51 (20.8%)		42 (29.0%)	
B2		15 (3.8%)		10 (4.1%)		5 (3.4%)	
B3		19 (4.9%)		13 (5.3%)		6 (4.1%)	
Radial nerve palsy at presentation	390	16 (4.1%)	245	13 (5.3%)	145	3 (2.1%)	0.185
Additional injuries	390	37 (9.5%)	245	26 (10.6%)	145	11 (7.6%)	0.374
Ipsilateral arm	390	7 (1.8%)	245	6 (2.4%)	145	1 (0.7%)	0.266
Contralateral arm	390	5 (1.3%)	245	4 (1.6%)	145	1 (0.7%)	0.655
Admission and follow-up characteristics							
Hospital							
Admission	390	271 (69.5%)	245	245 (100.0%)	145	26 (17.9%)	** < 0.001**
LOS (days)	271	2 (2–4)	245	2 (2–4)	26	2 (2–3)	0.830
Discharge disposition							
Home	271	258 (95.2%)	245	235 (95.9%)	26	23 (88.5%)	0.093
Care hotel		8 (3.0%)		6 (2.4%)		2 (7.7%)	
Elderly care facility		2 (0.7%)		1 (0.4%)		1 (3.8%)	
Rehabilitation center		3 (1.1%)		3 (1.2%)		0 (0.0%)	
Other care facility admission	390	13 (3.3%)	245	8 (3.3%)	145	5 (3.4%)	1.000
Nursing home, LOS (days)	1	30 (30–30)	1	30 (30–30)	0	N.A	N.A
Care hotel, LOS (days)	7	10 (5–30)	4	8 (5–25)	3	21 (3–21)	0.721
Elderly care facility, LOS (days)	4	35 (23–84)	1	21 (21–21)	3	42 (28–42)	0.180
Rehabilitation clinic, LOS (days)	3	25 (24–25)	3	25 (24–25)	0	N.A	N.A
Physical therapy (number of sessions)	337	24 (12–45)	217	25 (13–48)	120	22 (12–42)	0.307

*P*-values <0.05 are shown in boldface

Data are presented as *N* (%) or median (*P*_25_–*P*_75_)

*ASA* American Society of Anesthesiologists' classification, *BMI* body mass index, *LOS* length of stay

*N represents the number of patients for whom data were available per follow-up moment

### Treatment details and hospital admission

Osteosynthesis was performed by 121 surgeons, with 74 surgeons performing only one operation, and seven surgeons performing between five and 13 operations. Surgery was performed after a median of 6 (*P*_25_–*P*_75_ 2–9) days, with a median duration of surgery of 81 (*P*_25_–*P*_75_ 65–112) minutes. Intramedullary nailing was used in most patients (*N* = 169; 69.0%). In 158 (93.5%) of them, an antegrade nail was used. Seventy-six (31.0%) patients were treated using plate fixation. After a median stay of 2 (*P*_25_–*P*_75_ 2–4) days, the vast majority of operated patients (*N* = 235; 95.6%) were discharged home.

Fracture immobilization in the nonoperative group was performed using a brace (*N* = 68; 46.9%) or cast (*N* = 21; 14.5%). In 56 (38.6%), only a sling or collar and cuff were used. Twenty-six (17.9%) patients required hospital admission (Table 1). After a median stay of 2 (*P*_25_–*P*_75_ 2–3) days, most patients (*N* = 23; 88.5%) were discharged home. Hospital stay and subsequent stay in a nursing home, care hotel, elderly care facility, or rehabilitation center did not differ significantly between the two treatment groups. Likewise, patient in both groups had a similar number of physical therapy sessions; 217 (88.6%) and 120 (82.8%) patients in the operative and nonoperative group had physical therapy, respectively.

### Patient-reported functional outcome, pain, and activity resumption

**Fig. 2:**
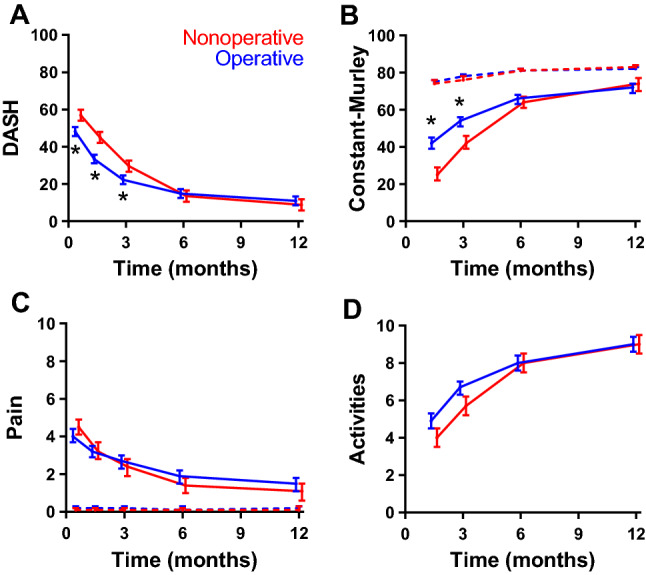
Changes in functional outcome scores, pain, and activity resumption over time by treatment group. **A** Disabilities of the Arm, Shoulder, and Hand (DASH) score, **B** Constant–Murley score of the affected arm, **C** pain (VAS, Visual Analog Scale) of the affected side, **D** the extent to which patients resumed their activities at the pre-trauma level (Numeric Rating Scale, NRS) over time. Higher scores represent more disability (DASH), a better function (Constant-Murley), more pain (VAS), and level of activity resumption (NRS, Numeric Rating Scale). Data are shown as estimated marginal mean with the corresponding 95% confidence interval, adjusted for age, gender, and fracture type, as emerging from the multivariable analysis. Blue lines represent the operative group; red lines represent the nonoperative group. In panel **C**, the dashed lines represent the contralateral side. *p < 0.05 (Bonferroni test).

The DASH (primary outcome measure), Constant–Murley, pain scores, and ability to perform daily activities improved over time in both the operative and nonoperative group (Fig. 2). Table 2 shows the results of the multilevel model, *i.e.*, the statistical significance of treatment effect and the estimated marginal means at three months; at that time a difference between the groups was expected. Supplemental Table S1 shows the original, unadjusted values (median, *P*_25_–*P*_75_, and univariate *p*-value) as well as the adjusted values (estimated marginal means with 95% CI) for all follow-up visits. The mean DASH score diminished from 48.2 points at two weeks to 11.0 points at 12 months in the operative group, and from 56.9 to 8.8 points in the nonoperative group (Fig. 2A). Patients in the operative group reported statistically significantly lower levels of disability until three months follow-up than patients in the nonoperative group. The interaction between treatment and time was also significant (*p*_interaction_ < 0.001); this reflects a difference in recovery speed between the two groups and the overlap in DASH values from three months onwards.

**Table 2:** Treatment effect over time and outcome at three months follow-up by treatment group

	Effect		Outcome at three months follow-up	
	*F* (*p*_treatment_)	*F* (*p*_interaction_)	Operative (*N* = 245)	Nonoperative (*N* = 145)
Patient-reported outcome measures				
DASH	11.79 (**0.001**)	27.30 (**< 0.001**)	**22.3 (19.9–24.6)**	**29.6 (26.6–32.6)**
Constant–Murley	19.45 (**< 0.001**)	44.23 (**< 0.001**)	**54 (51–56)**	**42 (39–46)**
Pain	0.50 (0.479**)**	3.98 (**0.003)**	2.7 (2.3–3.0)	2.4 (1.9–2.8)
Activity resumption	3.69 (0.056)	7.79 (**< 0.001**)	**6.7 (6.3–7.0)**	**5.7 (5.2–6.2)**
HR-QoL				
SF-36				
PCS	0.49 (0.484)	7.36 (**< 0.001**)	43 (42–44)	41 (40–43)
MCS	7.80 (**0.005**)	1.80 (0.126)	55 (54–57)	51 (49–53)
EQ-5D				
US	6.04 (**0.014**)	10.97 (**< 0.001**)	0.77 (0.74–0.80)	0.72 (0.68–0.75)
VAS	0.96 (0.328)	1.73 (0.141)	76 (74–78)	74 (72–77)
Shoulder range of motion (°)				
Abduction	45.79 (**< 0.001**)	43.01 (**< 0.001**)	**105 (99–110)**	**77 (70–84)**
Anteflexion	67.27 (**< 0.001**)	70.32 (**< 0.001**)	**111 (106–116)**	**81 (75–88)**
External rotation	156.03 (**< 0.001**)	81.58 (**< 0.001**)	**58 (54–61)**	**41 (36–45)**
Internal rotation	0.32 (0.570)	0.64 (0.636)	58 (55–60)	57 (53–60)
Elbow range of motion (°)				
Flexion	52.86 (**< 0.001**)	21.11 (**< 0.001**)	**137 (135–139)**	**129 (126–132)**
Extension deficit	56.41 (**< 0.001**)	27.60 (**< 0.001**)	**− 5 (− 7 to − 3)**	**− 11 (− 14 to − 8)**
Pronation	42.88 (**< 0.001**)	16.99 (**< 0.001**)	84 (82–85)	81 (79–83)
Supination	28.64 (**< 0.001**)	22.68 (**< 0.001**)	82 (80–84)	78 (76–881)

Changes in recovery pattern were assessed in the multilevel model. Results are shown by the *F *value for treatment and for the interaction term in the model (treatment * follow-up moment), with their corresponding *p* value in parenthesis

*DASH* disabilities of the arm, shoulder, and hand, *EQ-5D* EuroQoL-5D, *HR-QoL* health-related quality of life, *MCS* mental component summary, *PCS* physical component summary, *SF-36* short form-36, *US* utility score, *VAS* visual analog scale

Data of the outcome at three months are shown as the estimated marginal mean with 95% confidence interval after three months follow-up adjusted for age, gender, and fracture type. If the intervals did not overlap, this is indicated in bold face. The Constant–Murley score, pain score, and ranges of motion of the shoulder and arm are shown for the affected side. Bold face indicates that the 95% confidence interval of the two treatment groups did not overlap (*p* < 0.05, Bonferroni test)

Similar as for the DASH, the Constant–Murley score also showed a significant treatment effect in favor of the operative group (*p*_treatment_ < 0.001). Patients in this group also recovered faster (*p*_interaction_ < 0.001; Fig. 2B and Table 2). Scores for the affected side increased from 42 points at six weeks to 72 points at 12 months in the operative group and from 25 to 74 points in the nonoperative group (Fig. 2B). Significantly higher scores for the affected side were noted in the operative group at 6 weeks (42 versus 25 points) and three months (54 versus 42), but not at later time points. The values at the contralateral side stayed consistently between 74 and 83 in both groups.

The course of pain was not significantly associated with treatment (*p*_treatment_ = 0.479; Fig. 2C). The total reduction in pain level was, however, slightly more pronounced in the nonoperative group (*p*_interaction_ = 0.003). Patients reported no pain at the contralateral side.

Patients in the operative group reported a better ability to participate in activities like sports and hobbies at six weeks (4.9 versus 4.0 in the nonoperative group) and three months (6.7 versus 5.7), yet both groups reported 9.0 at 12 months. This resulted in a significant interaction (*p*_interaction_ < 0.001), but the overall treatment effect was non-significant (*p*_treatment_ = 0.056).

### Health-related quality of life

**Fig. 3:**
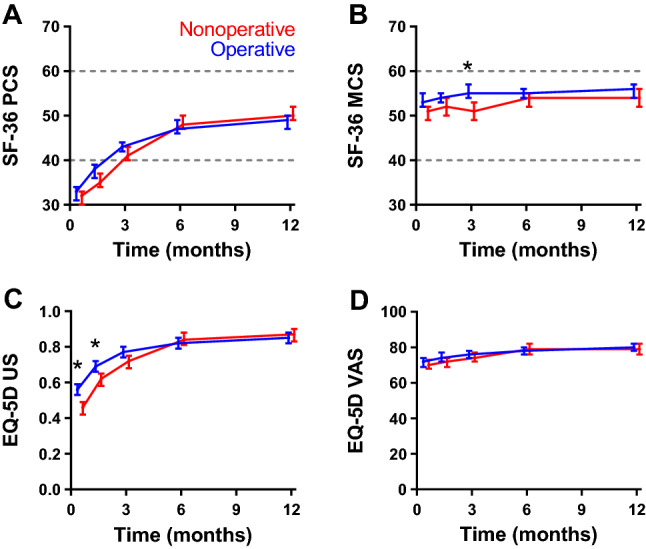
Changes in health-related quality of life over time by treatment group. **A** Short Form-36 (SF-36) Physical Component Summary (SF-36 PCS), **B** SF-36 Mental Component Summary (SF-36 MCS), **C** EuroQoL-5D-3L (EQ-5D) utility score (EQ-5D US), and **D** EQ-5D Visual Analog Scale (EQ-5D VAS) over time. Higher scores represent better quality of life. Data are shown as estimated marginal mean with the corresponding 95% confidence interval, adjusted for age, gender, and fracture type, as emerging from the multivariable analysis. Blue lines represent the operative group; red lines represent the nonoperative group. In panels **A** and **B**, the dashed lines represent the mean ± SD (50 ± 10) that was used for normalizing the data. *p < 0.05 (Bonferroni test)

Figure 3 shows changes in HR-QoL over time. The corresponding estimated marginal means at three months and results of the multilevel models are shown in Table 2 and Supplemental Table S1. The SF-36 PCS improved at similar speed over time in both groups, from 32 at two weeks to 50 at 12 months in the nonoperative group and from 33 to 49 in the operative group. From three months onwards, it was within the normal range of 50 ± 10 points. The SF-36 MCS was consistently within the normal range throughout the entire follow-up period, with the entire curve of the operative group being just above that of the nonoperative group (*p*_treatment_  = 0.005).

The EQ-5D US was significantly higher in the operative group at two and six weeks (0.56 and 0.69) than in the nonoperative group (0.46 and 0.62) and showed a significant treatment effect and interaction with time (*p*_treatment_ = 0.014 and *p*_interaction_ < 0.001). The EQ-VAS, on the other hand, was unaffected by the type of treatment and hardly improved over time (*p*_treatment_ = 0.328 and *p*_interaction_ = 0.141).

### Range of motion

**Fig. 4:**
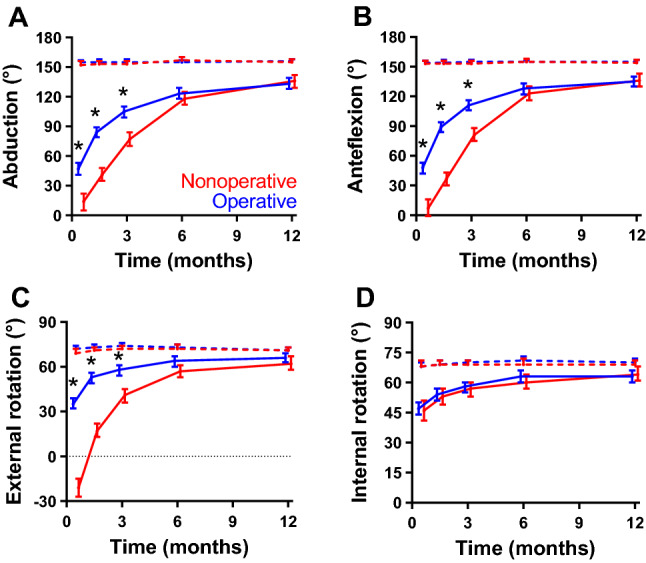
Changes in range of motion of the shoulder over time by treatment group. **A** Abduction, **B** anteflexion, **C** external rotation, and **D** internal rotation of the shoulder over time. Higher scores represent better range of motion (ROM). Data are shown as estimated marginal mean with the corresponding 95% confidence interval, adjusted for age, gender, and fracture type, as emerging from the multivariable analysis. Blue lines represent the operative group; red lines represent the nonoperative group. Dashed lines represent the contralateral side. *p < 0.05 (Bonferroni test)

Changes in ROM of the shoulder are shown in Fig. 4, Table 2, and Supplemental Table S1. Abduction, anteflexion, and external rotation of the shoulder all showed a significant treatment effect and interaction with time (*p*_treatment_ < 0.001 and *p*_interaction_ < 0.001). For all three motions, the values were between 33 and 56° higher in the operative group than in the nonoperative group. The largest difference was seen for external rotation at two weeks; 35° in the operative group versus − 21° in the nonoperative group. The difference reduced over time but remained statistically significant until three months follow-up. Treatment had no significant effect on internal rotation (*p*_treatment_ = 0.571 and *p*_interaction_ = 0.636).

**Fig. 5:**
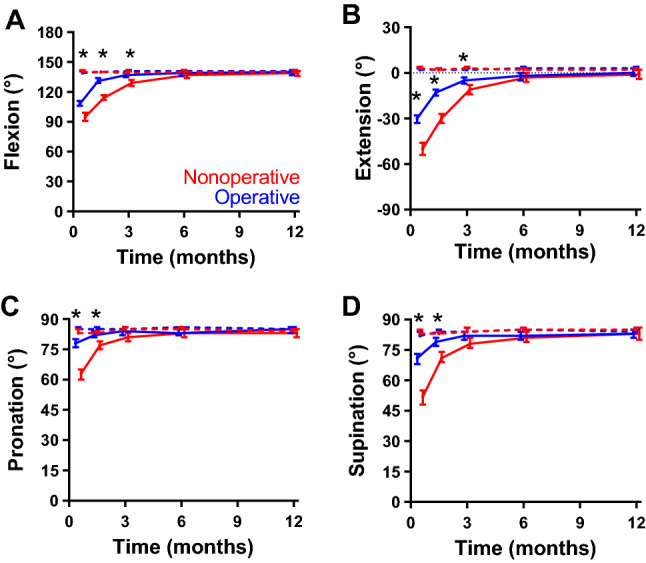
Changes in range of motion of the elbow over time by treatment group. **A** Flexion, **B** extension, **C** pronation, and **D** supination of the elbow over time. Higher scores represent better range of motion (ROM). Data are shown as estimated marginal mean with the corresponding 95% confidence interval, adjusted for age, gender, and fracture type, as emerging from the multivariable analysis. Blue lines represent the operative group; red lines represent the nonoperative group. *p < 0.05 (Bonferroni test)

Changes in ROM of the elbow are shown in Fig. 5, Table 2 and Supplemental Table S1. All measured ranges of motion of the elbow were statistically significantly better for the operated patients than for the nonoperated patients until six week follow-up (pronation and supination) or three months follow-up (flexion and extension). All ranges of motion of the elbow recovered to about the same values as the contralateral side and showed a significant treatment effect and interaction with time (*p*_treatment_ < 0.001 and *p*_interaction_ < 0.001).

### Resumption of work and sports

Table 3 shows the patients’ participation and resumption of work and sports. About half of the patients (*N* = 198) had a paid job prior to their injury. Paid work was significantly more common in the operative group (*N* = 136; 55.5%) than in the nonoperative group (*N* = 62; 42.8%; *p* = 0.016). These patients also worked more hours per week (38 versus 32; *p* = 0.016). The exertional level was similar in both groups. Work absenteeism post-injury was reported by more than 90% of patients. Although the operative group resumed work seven work days earlier (26 days versus 33 in the nonoperative group), this did not reach statistical significance (*p*  = 0.253).

**Table 3:** Work and sports participation pre-trauma and post-trauma resumption of study participants by treatment group

	N*	All (*N* = 390)	N*	Operative (*N* = 245)	N*	Nonoperative (*N* = 145)	*P* value
**Work**							
Paid work (*N* patients)	390	198 (50.8%)	245	136 (55.5%)	145	62 (42.8%)	**0.016**
Hours per week	194	36 (27–40)	134	38 (32–40)	60	32 (21–40)	**0.016**
Exertional level							
Light, mainly sedentary	198	88 (44.4%)	136	64 (47 1%)	62	24 (38.7%)	0.304
Medium work		71 (35.9%)		49 (36.0%)		22 (35.5%)	
Heavy or very heavy work		39 (19.7%)		23 (16.9%)		16 (25.8%)	
Work absence	196	179 (91.3%)	134	123 (91.8%)	62	56 (90.3%)	0.787
Work days missed	196	30 (13–54)	134	26 (12–49)	62	33 (15–59)	0.253
Sports or hobby							
Sports or hobby (N patients)	390	384 (98.5%)	245	242 (98.8%)	145	142 (97.9%)	0.675
Hours per week	378	17 (9–31)	240	16 (9–30)	138	19 (9–32)	0.115
Resumption at 12 months	241	340 (99.7%)	215	214 (99.5%)	126	126 (100.0%)	1.000

*P*-values <0.05 are shown in boldface

Data are presented as *N* (%) or median (*P*_25_–*P*_75_)

*N represents the number of patients for whom data were available per follow-up moment.

Overall, 378 (98.5%) patients participated in sports or hobbies pre-trauma, for a median of 17 h per week, all but one patient resumed sports and hobbies during follow-up. No significant differences were noted between the two treatment groups.

### Complications and secondary surgical interventions

Complications were more common in the nonoperative group (*N* = 50; 34.5%) than in the operative group (*N* = 58; 23.7%; *p* = 0.026; Table 4). As a consequence, secondary surgical interventions were also done more frequently in the nonoperative group (*N* = 37 (25.5%) versus *N* = 20 (12.2%); *p* = 0.001). Malalignment occurred only in the nonoperative group (*N* = 14; 9.7%); 11 of these patients were operated. In the operative group, implant-related complications were most common (*N* = 26; 10.6%). This included nail protrusion (*N* = 13), screw protrusion (*N* = 8), screw cutout (*N* = 2), inadequate implant size (*N* = 1) or implant type (*N* = 1), or chronic pain (*N* = 1). These complications resulted in implant exchange or removal in three and 16 patients, respectively. Five nonoperatively treated patients developed disproportionate pain, resulting in secondary osteosynthesis. Postoperative or persistent radial nerve palsy, which occurred in nine (3.7%) patients of the operative group and three (2.1%) patients of the nonoperative group, fully recovered in 86% and 67% of patients, respectively (*p* = 0.437). Nonunion occurred significantly more often in the nonoperative group (*N* = 30; 26.3%) than in the operative group (*N* = 19; 10.1%; *p* < 0.001). Twenty of these 30 and 10 of the 19 patients underwent (revision) osteosynthesis within a year after injury.

**Table 4:** Complications and associated secondary surgical intervention by treatment group

	N*	All (*N* = 390)	N*	Operative (*N* = 245)	N*	Nonoperative (*N* = 145)	*P* value
Any complication	390	108 (27.7%)	245	58 (23.7%)	145	50 (34.5%)	**0.026**
Any surgical re-intervention	390	67 (17.2%)	245	30 (12.2%)	145	37 (25.5%)	**0.001**
Malalignment	390	14 (3.6%)	245	0 (0.0%)	145	14 (9.7%)	** < 0.001**
Osteosynthesis		11		N.A.		11	N.A.
Cuff pathology	390	3 (0.8%)	245	3 (1.2%)	145	0 (0.0%)	0.298
Superficial infection	390	6 (1.5%)	245	5 (2.0%)	145	1 (0.7%)	0.419
Deep infection	390	1 (0.3%)	245	1 (0.4%)	145	0 (0.0%)	1.000
Drainage and implant removal		1		1		N.A.	N.A.
Implant-related complication	390	26 (6.7%)	245	26 (10.6%)	145	0 (0.0%)	** < 0.001**
Nail protrusion		13		13		N.A.	
Screw protrusion		8		8		N.A.	
Screw cutout		2		2		N.A.	
Inadequate implant size		1		1		N.A.	
Inadequate implant type		1		1		N.A.	
Chronic pain		1		1		N.A.	
Implant exchange		3		3		N.A.	N.A.
Implant removal		16		16		N.A.	N.A.
Disproportional pain and disability	390	5 (1.3%)	245	0 (0.0%)	145	5 (3.4%)	**0.007**
Osteosynthesis		5		N.A		5	N.A.
Post-operative or persistent radial nerve apraxia	390	12 (3.3%)	245	9 (3.7%)	145	3 (2.1%)	N.A.
Osteosynthesis		1		0		1	N.A.
Osteosynthesis and nerve grafting		1		0		1	N.A.
Malunion	390	3 (0.8%)	245	0 (0.0%)	145	3 (2.1%)	0.051
Nonunion	302	49 (16.2%)	188	19 (10.1%)	114	30 (26.3%)	** < 0.001**
(Revision) osteosynthesis		30		10		20	

*P*-values <0.05 are shown in boldface

Data are presented as number (%) or as median (*P*_25_–*P*_75_) and were analyzed using a Chi-squared test and Mann–Whitney *U* test, respectively

*N represents the number of patients for whom data were available per follow-up moment

## Discussion

Data from the current multicenter prospective study demonstrate that adult patients with a closed humeral shaft fracture AO type 12A or 12B treated operatively have a better outcome until six months than patients treated nonoperatively in terms of a lower DASH score, higher Constant–Murley score, improved shoulder and elbow ROM, and a higher health-related quality of life (EQ-5D US). In addition, the operated group had fewer complications and surgical re-interventions. Given the multicenter design, the findings of this study can be generalized and therefore will apply to all different levels of trauma centers.

The statistically significant difference in DASH score in the first six months after trauma of 8.8 points or more in favor of the operative group is in line with previous RCTs which show a mean difference of 18.0 and 6.0 points at six months [23, 24]. In addition, the FISH trial also shows superior DASH scores until six month follow-up [25]. The differences are larger than the minimally important change for the DASH (6.7 points) in the study population, confirming that our findings are statistically as well as clinically significant [20]. Quick-DASH correlates highly with function and patient satisfaction, and is considered a suitable tool for evaluating adult humeral shaft outcomes [26].

Similar as the DASH, the Constant–Murley score also showed superior upper extremity function in the operative group until six months after trauma. This was also shown in the FISH trial [25], however, another RCT by Matsunaga et al*.* found no significant difference in score during a 12 month follow-up period [24]. It is not clear if this lack of difference can be attributed to a lower mean age, lower proportion of females, and inclusion of 12% of patients with an AO type 12C fractures in the nonoperative group in their study.

With regards to complications, both the current data and a meta-analysis show that pain, infection, and radial nerve palsy are no contributing factors in the decision-making for humeral shaft fractures [2]. Both operatively and nonoperatively treated patients in the current study reported a similar level and decrease of pain during the 12 month follow-up. Similar findings have previously also been reported [24]. Rämö et al*.*, on the other hand, reported slightly, yet statistically significant, less pain in the operative group until six weeks after trauma, but the difference in pain was less than the threshold for clinical relevance [25]. In any case, pain per se is no contra-indication for operative management. In fact, five (3.4%) patients in the nonoperative group of the current study were operated due to disproportional pain.

Six patients out of 245 operated patients in our study had an infection (2.4%), of which five were only superficial according to the CDC classification. This is slightly less than the 3.1% out of 611 operated patients as reported in a recent meta-analysis [2].

Sixteen (4.1%) patients presented with radial nerve palsy after trauma, which is a much lower rate than the 15.6% (201/1,289) reported in a meta-analysis [2]. The postoperative radial nerve palsy rate in their study was 3.6%, with a full recovery rate (at follow-up ranging from 6 to 72 months) of 96.4%. In our study, nine of out 232 (3.9%) patients developed a postoperative radial nerve palsy, of whom eight showed full recovery within the 12 month follow-up. This implies that the risk of persistent radial nerve palsy due to surgery at 12 months is 0.4% (*i.e.*, 1/232), and this minimal risk should be no reason to avoid surgery.

An inherent disadvantage of operative management is the risk of implant-related complications. Implant removal was performed due to nail or screw protrusion or chronic pain in 16/245 (6.9%) patients who were all treated with an IMN. For the same indication, hardware removal was reported in 10/156 (6.4%) patients in one RCT and three observational studies [23, 27–29].

To achieve early functional recovery, treatment should focus on timely fracture healing and preventing malalignment. In this study, malalignment only occurred after nonoperative treatment, with 11 out of 14 patients requiring revision surgery. This rate of 9.7% is in line with 11.0% as calculated from one RCT and three observational studies [24, 27, 28, 30]. The risk of nonunion in our study was 2.6-fold higher after nonoperative treatment than after operative treatment (*i.e.*¸ 26.3% versus 10.1%). Analogous to our data, another RCT and two observational studies show a 2–2.5-fold higher nonunion rate after nonoperative treatment [23, 31, 32]. The effect was even stronger in two RCTs, which show 15 and 25% nonunion in the nonoperative group versus none at all after surgery [24, 25]. With data supporting that nonunion can, to a large extent, be prevented by immediate surgery, surgery should be the first option for the treatment of humeral shaft fractures.

### Strengths and limitation

The main strength of this prospective, multicenter study is that it is the largest series of patients with a humeral shaft fracture to date. The sample size was much higher than 47 to 110 patients in the most recent prospective studies on this topic [24–26, 33, 34]. Combined with the participation of 29 hospitals across the country, including level 1, 2, and 3 trauma centers, it therewith represents current practice. Furthermore, treatment heterogeneity across participating hospitals caused by not standardizing treatment or rehabilitation will improve generalization of the results. The higher prevalence of females and higher median age in the nonoperative group in this study is in line with published data [2]. This may also explain the higher prevalence of osteoporosis/osteopenia in the nonoperative group. Overall, this indicates that selection bias due to the study design is unlikely, based on these patient characteristics.

A benefit of the observational design, allowing surgeon to decide on treatment, surgical approach, and implant, is that surgeons could use the (operative) technique they felt was best for the individual patient in their hands. This in contrast to a randomized design where randomization could result in the (operative) technique where the surgeon would feel less comfortable with or had less experience in. Another strength is that dedicated researchers performed the follow-up measurements of all patients. This centralized coordination allowed hospitals with insufficient research resources to participate. In a previous study it was shown that data quality and completeness can benefit from central study coordination [35].

As commonly seen in observational studies, some imbalance in baseline data was noted between the two treatment groups. Although this may be considered as a limitation, we were able to correct for this in the mixed-linear models. When designing the study, we considered a RCT not feasible. The rationale, which includes strong patient and surgeon preference and early termination of RCTs at that time due to enrollment issues, is elaborated on the published study protocol [12].Another limitation could be that some participating hospitals enrolled < 5 patients, suggesting that not all patients were screened for participation. Overall, 46 patients were missed for screening or declined participation. Consequently, the study sample was not consecutive. As this study did not interfere with treatment decision, it is unlikely that this has introduced selection bias or affected validity of the results. On the other hand, despite great efforts of the researchers, some bias due to missed follow-up visits and consent withdrawal cannot be ruled out. As this was the case in 19% of patients in both treatment arms, this is unlikely to be differential.

## Conclusion

Primary osteosynthesis of a humeral shaft fracture (AO type 12A and 12B) in adults is safe and superior to nonoperative treatment, and should therefore be the treatment of choice. It is associated with a more than twofold reduced risk of nonunion, earlier functional recovery and a better range of motion of the shoulder and elbow joint than nonoperative treatment. Even after including the implant-related complications, the overall rate of complications as well as secondary surgical interventions was highest in the nonoperative group.

## Supplementary Information

Below is the link to the electronic supplementary material.

Supplementary file1 (DOCX 32 kb)

## Data Availability

No additional data are available. Data can be made available upon reasonable request to the principal investigator.

## References

[1] Ekholm R, Adami J, Tidermark J, Hansson K, Tornkvist H, Ponzer S. Fractures of the shaft of the humerus. An epidemiological study of 401 fractures. J Bone Joint Surg Br. 2006;88(11):1469–73. 10.1302/0301-620X.88B11.17634 17075092

[2] Van de Wall BJM, Ochen Y, Beeres FJP, Babst R, Link BC, Heng M, et al. Conservative vs. operative treatment for humeral shaft fractures: a meta-analysis and systematic review of randomized clinical trials and observational studies. J Shoulder Elbow Surg. 2020;29(7):1493–504. 10.1016/j.jse.2020.01.072 32249144

[3] Schittko A. [Humeral shaft fractures]. Chirurg. 2004;75(8):833–46; quiz 47. 10.1007/s00104-004-0911-z 15278235

[4] Volgas DA, Stannard JP, Alonso JE (2004). Nonunions of the humerus. Clin Orthop Relat Res.

[5] Sarmiento A, Zagorski JB, Zych GA, Latta LL, Capps CA (2000). Functional bracing for the treatment of fractures of the humeral diaphysis. J Bone Joint Surg Am.

[6] Toivanen JA, Nieminen J, Laine HJ, Honkonen SE, Jarvinen MJ (2005). Functional treatment of closed humeral shaft fractures. Int Orthop.

[7] Shao YC, Harwood P, Grotz MR, Limb D, Giannoudis PV (2005). Radial nerve palsy associated with fractures of the shaft of the humerus: a systematic review. J Bone Joint Surg Br.

[8] Mahabier KC, Vogels LMM, Punt BJ, Roukema GR, Patka P, Van Lieshout EMM (2013). Humeral shaft fractures: retrospective results of non-operative and operative treatment of 186 patients. Injury.

[9] Gosler MW, Testroote M, Morrenhof JW, Janzing HM. Surgical versus non-surgical interventions for treating humeral shaft fractures in adults. Cochrane Database Syst Rev. 2012;1:CD008832. 10.1002/14651858.CD008832.pub2 PMC12186995 22258990

[10] Kwaees TA, Zreik NH, Charalambous CP. Surgical vs. nonsurgical management for humeral shaft fractures; preference among orthopaedic surgeons. Ortop Traumatol Rehabil. 2021;23(1):21–6. 10.5604/01.3001.0014.7564 33709952

[11] Fracture and dislocation compendium. Orthopaedic Trauma Association Committee for Coding and Classification. J Orthop Trauma. 1996;10(Suppl 1:v-ix), 1–154. 8814583

[12] Mahabier KC, Van Lieshout EMM, Bolhuis HW, Bos PK, Bronkhorst MWGA, Bruijninckx MMM (2014). HUMeral shaft fractures: measuring recovery after operative versus non-operative treatment (HUMMER): a multicenter comparative observational study. BMC Musculoskelet Disord.

[13] Beaton DE, Katz JN, Fossel AH, Wright JG, Tarasuk V, Bombardier C (2001). Measuring the whole or the parts? Validity, reliability, and responsiveness of the disabilities of the arm, shoulder and hand outcome measure in different regions of the upper extremity. J Hand Ther.

[14] Hudak PL, Amadio PC, Bombardier C. Development of an upper extremity outcome measure: the DASH (disabilities of the arm, shoulder and hand) [corrected]. The Upper Extremity Collaborative Group (UECG). Am J Ind Med. 1996;29(6):602–8. 10.1002/(SICI)1097-0274(199606)29:6<602::AID-AJIM4>3.0.CO;2-L 8773720

[15] Constant CR, Murley AH (1987). A clinical method of functional assessment of the shoulder. Clin Orthop Relat Res.

[16] Brooks R, Rabin RE, Eds. DC. The measurement and valuation of health status using EQ-5D: a European perspective. Kluwer Academic Publishers. 2003.

[17] Lamers LM, Stalmeier PF, McDonnell J, Krabbe PF, van Busschbach JJ (2005). Measuring the quality of life in economic evaluations: the Dutch EQ-5D tariff. Ned Tijdschr Geneeskd.

[18] Ware JE, Jr., Sherbourne CD. The MOS 36-item short-form health survey (SF-36). I. Conceptual framework and item selection. Med Care. 1992;30(6):473–83. 1593914

[19] Anglen JO, Archdeacon MT, Cannada LK, Herscovici D (2008). Avoiding complications in the treatment of humeral fractures. J Bone Joint Surg Am.

[20] Mahabier KC, Den Hartog D, Theyskens N, Verhofstad MHJ, Van Lieshout EMM, Investigators HT (2017). Reliability, validity, responsiveness, and minimal important change of the Disabilities of the Arm, Shoulder and Hand and Constant-Murley scores in patients with a humeral shaft fracture. J Shoulder Elbow Surg.

[21] Van Lieshout EMM, Mahabier KC, Tuinebreijer WE, Verhofstad MHJ, Den Hartog D, Investigators H. Rasch analysis of the Disabilities of the Arm, Shoulder and Hand (DASH) instrument in patients with a humeral shaft fracture. J Shoulder Elbow Surg. 2019. 10.1016/j.jse.2019.09.026 31786010

[22] Ekholm R, Ponzer S, Tornkvist H, Adami J, Tidermark J (2008). Primary radial nerve palsy in patients with acute humeral shaft fractures. J Orthop Trauma.

[23] Kumar S, Shanmugam N, Kumar S, Ramanusan R (2017). Comparison between operative and non operative treatment of fracture shaft of humerus: an outcome analysis. Int J Res Orthop.

[24] Matsunaga FT, Tamaoki MJ, Matsumoto MH, Netto NA, Faloppa F, Belloti JC (2017). Minimally invasive osteosynthesis with a bridge plate versus a functional brace for humeral shaft fractures: A randomized controlled trial. J Bone Joint Surg Am.

[25] Ramo L, Sumrein BO, Lepola V, Lahdeoja T, Ranstam J, Paavola M (2020). Effect of surgery vs functional bracing on functional outcome among patients with closed displaced humeral shaft fractures: The FISH randomized clinical trial. JAMA.

[26] Belangero WD, Zublin CM, Quintero RAC, Romero FAS, Fernandes HJA, Siekavizza SNM (2020). Quick-DASH as a main early outcome of humeral shaft fractures: A Latin American multicenter prospective study. J Orthop Surg (Hong Kong).

[27] Jawa A, McCarty P, Doornberg J, Harris M, Ring D. Extra-articular distal-third diaphyseal fractures of the humerus. A comparison of functional bracing and plate fixation. J Bone Joint Surg Am. 2006;88(11):2343–7. 10.2106/JBJS.F.00334 17079389

[28] Osman N, Touam C, Masmejean E, Asfazadourian H, Alnot JY. Results of non-operative and operative treatment of humeral shaft fractures. A series of 104 cases. Chir Main. 1998;17(3):195–206. 10.1016/s0753-9053(98)80039-2 10855286

[29] Wallny T, Sagebiel C, Westerman K, Wagner UA, Reimer M (1997). Comparative results of bracing and interlocking nailing in the treatment of humeral shaft fractures. Int Orthop.

[30] Dielwart C, Harmer L, Thompson J, Seymour RB, Karunakar MA (2017). Management of closed diaphyseal humerus fractures in patients with injury severity score >/=17. J Orthop Trauma.

[31] Denard A, Jr., Richards JE, Obremskey WT, Tucker MC, Floyd M, Herzog GA. Outcome of nonoperative vs operative treatment of humeral shaft fractures: a retrospective study of 213 patients. Orthopedics. 2010;33(8). 10.3928/01477447-20100625-16 20704103

[32] Westrick E, Hamilton B, Toogood P, Henley B, Firoozabadi R (2017). Humeral shaft fractures: results of operative and non-operative treatment. Int Orthop.

[33] Akalin Y, Sahin IG, Cevik N, Guler BO, Avci O, Ozturk A (2020). Locking compression plate fixation versus intramedullary nailing of humeral shaft fractures: which one is better? A single-centre prospective randomized study. Int Orthop.

[34] Van Middendorp JJ, Kazacsay F, Lichtenhahn P, Renner N, Babst R, Melcher G (2011). Outcomes following operative and non-operative management of humeral midshaft fractures: a prospective, observational cohort study of 47 patients. Eur J Trauma Emerg Surg.

[35] Zielinski SM, Viveiros H, Heetveld MJ, Swiontkowski MF, Bhandari M, Patka P (2012). Central coordination as an alternative for local coordination in a multicenter randomized controlled trial: the FAITH trial experience. Trials.

