# Supplementary material for: Functional and clinical outcome after operative versus nonoperative treatment of a humeral shaft fracture (HUMMER): results of a multicenter prospective cohort study
Source: Eur J Trauma Emerg Surg. 2022 Feb 9;48(4):3265–77. doi: 10.1007/s00068-022-01890-6 (PMC9360107; doi:10.1007/s00068-022-01890-6)
Supplement: Supplementary file 1 — Supplementary file1 (DOCX 32 kb) [file 68_2022_1890_MOESM1_ESM.docx]

**Supplemental Table S1. Functional outcome and range of motion of the shoulder and elbow over time by treatment group, based upon univariate and multivariable repeated measure analysis**

|  | **Time** |  | | **Univariate analysis** | | | | | **Multivariable, repeated measure analysis** | | |
| --- | --- | --- | --- | --- | --- | --- | --- | --- | --- | --- | --- |
|  |  | **All**  **(N=390)** | | **Operative**  **(N=245)** | | **Nonoperative**  **(N=145)** | | **P-value** | **Operative**  **(N=245)** | **Nonoperative**  **(N=145)** |  |
|  |  | **N*** |  | **N*** |  | **N*** |  |  |  |  |  |
| DASH | 2 we | 360 | 51.7 (37.7-63.3) | 227 | 48.3 (33.9-58.3) | 133 | 59.2 (48.3-67.5) | **<0.001** | **48.2 (45.8-50.5)** | **56.9 (53.9-59.9)** |  |
|  | 6 we | 366 | 35.8 (21.7-52.5) | 229 | 29.2 (17.5-43.3) | 137 | 48.3 (33.3-58.8) | **<0.001** | **33.4 (31.1-35.7)** | **45.0 (42.1-48.0)** |  |
|  | 3 mo | 348 | 20.8 (10.1-34.9) | 220 | 17.5 (7.5-30.6) | 128 | 26.3 (15.2-43.3) | **<0.001** | **22.3 (19.9-24.6)** | **29.6 (26.6-32.6)** |  |
|  | 6 mo | 337 | 8.3 (2.5-20.3) | 209 | 8.3 (2.5-18.3) | 128 | 9.6 (1.7-22.3) | 0.468 | 14.9 (12.5-17.3) | 13.5 (10.5-16.5) |  |
|  | 12 mo | 344 | 3.3 (0.0-14.2) | 217 | 3.3 (0.0-14.2) | 127 | 3.3 (0.0-12.5) | 0.855 | 11.0 (8.6-13.3) | 8.8 (5.8-11.8) |  |
| Constant-Murley | 2 we | N.A. | N.A. | N.A. | N.A. | N.A. | N.A. | N.A. | N.A. | N.A. |  |
|  | 6 we | 339 | 33 (21-52) | 220 | 41 (27-58) | 119 | 21 (14-31) | **<0.001** | **42 (39-45)** | **25 (22-29)** |  |
|  | 3 mo | 344 | 51 (35-65) | 218 | 55 (39-72) | 126 | 40 (24-57) | **<0.001** | **54 (51-56)** | **42 (39-46)** |  |
|  | 6 mo | 331 | 69 (55-79) | 205 | 70 (56-82) | 126 | 66 (50-76) | **0.007** | 66 (63-68) | 64 (61-67) |  |
|  | 12 mo | 339 | 77 (65-83) | 213 | 77 (65-84) | 126 | 77 (67-82) | 0.553 | 72 (69-74) | 74 (70-77) |  |
| Pain (VAS) | 2 we | 359 | 3.7 (1.9-6.4) | 226 | 3.4 (1.9-5.7) | 133 | 4.4 (2.1-6.9) | 0.082 | 4.0 (3.7-4.4) | 4.5 (4.1-4.9) |  |
|  | 6 we | 364 | 2.6 (1.1-4.8) | 228 | 2.3 (0.9-5.0) | 136 | 3.0 (1.6-4.7) | 0.144 | 3.2 (2.9-3.5) | 3.2 (2.8-3.7) |  |
|  | 3 mo | 346 | 1.7 (0.5-4.0) | 220 | 1.5 (0.2-4.4) | 126 | 1.9 (0.8-3.6) | 0.393 | 2.7 (2.3-3.0) | 2.4 (1.9-2.8) |  |
|  | 6 mo | 336 | 0.8 (0.0-2.1) | 208 | 0.9 (0.0-2.1) | 128 | 0.5 (0.0-1.9) | 0.199 | 1.9 (1.5-2.2) | 1.4 (1.0-1.8) |  |
|  | 12 mo | 344 | 0.0 (0.0-1.5) | 217 | 0.0 (0.0-1.7) | 127 | 0.0 (0.0-1.3) | 0.649 | 1.5 (1.1-1.8) | 1.1 (0.6-1.5) |  |
| Activity resumption (NRS) | 2 we | N.A. | N.A. | N.A. | N.A. | N.A. | N.A. | N.A. | N.A. | N.A. |  |
|  | 6 we | 361 | 5 (3-7) | 228 | 5 (3-8) | 133 | 4 (2-6) | **0.007** | **4.9 (4.5-5.3)** | **4.0 (3.5-4.5)** |  |
|  | 3 mo | 348 | 7 (5-9) | 217 | 8 (5-9) | 131 | 6 (3-9) | **0.015** | **6.7 (6.3-7.0)** | **5.7 (5.2-6.2)** |  |
|  | 6 mo | 333 | 9 (7-10) | 207 | 9 (7-10) | 126 | 9 (7-10) | 0.549 | 8.0 (7.6-8.4) | 8.0 (7.5-8.5) |  |
|  | 12 mo | 336 | 10 (9-10) | 211 | 10 (9-10) | 125 | 10 (9-10) | 0.084 | 9.0 (8.6-9.4) | 9.0 (8.5-9.5) |  |
| SF-36 PCS | 2 we | 334 | 33 (28-37) | 214 | 34 (30-38) | 120 | 30 (25-37) | **0.001** | 33 (31-34) | 32 (30-33) |  |
|  | 6 we | 339 | 37 (32-42) | 210 | 38 (33-43) | 129 | 34 (29-39) | **<0.001** | 38 (36-39) | 35 (34-37) |  |
|  | 3 mo | 322 | 43 (36-50) | 207 | 44 (38-51) | 115 | 41 (36-47) | **0.002** | 43 (42-44) | 41 (40-43) |  |
|  | 6 mo | 319 | 50 (43-55) | 196 | 51 (43-55) | 123 | 50 (42-55) | 0.541 | 47 (46-49) | 48 (47-50) |  |
|  | 12 mo | 333 | 53 (45-57) | 211 | 52 (45-57) | 122 | 53 (44-56) | 0.989 | 49 (47-50) | 50 (49-52) |  |
| SF-36 MCS | 2 we | 334 | 54 (46-61) | 214 | 55 (47-61) | 120 | 52 (45-60) | 0.202 | 53 (52-55) | 51 (49-52) |  |
|  | 6 we | 339 | 56 (48-61) | 210 | 57 (49-61) | 129 | 54 (47-60) | 0.104 | 54 (53-55) | 52 (50-54) |  |
|  | 3 mo | 322 | 57 (49-61) | 207 | 58 (54-61) | 115 | 55 (44-60) | **0.002** | **55 (54-57)** | **51 (49-53)** |  |
|  | 6 mo | 319 | 57 (53-60) | 196 | 57 (54-60) | 123 | 56 (50-61) | 0.449 | 55 (54-56) | 54 (52-55) |  |
|  | 12 mo | 333 | 58 (53-60) | 211 | 58 (53-60) | 122 | 57 (51-60) | 0.198 | 56 (54-57) | 54 (52-56) |  |
| EQ-5D US | 2 we | 361 | 0.55 (0.40-0.73) | 228 | 0.67 (0.43-0.73) | 133 | 0.43 (0.31-0.67) | **<0.001** | **0.56 (0.53-0.59)** | **0.46 (0.42-0.49)** |  |
|  | 6 we | 364 | 0.73 (0.56-0.81) | 228 | 0.73 (0.61-0.84) | 136 | 0.69 (0.47-0.73) | **<0.001** | **0.69 (0.66-0.72)** | **0.62 (0.58-0.65)** |  |
|  | 3 mo | 348 | 0.81 (0.69-0.90) | 220 | 0.81 (0.73-0.90) | 128 | 0.73 (0.61-0.84) | **<0.001** | 0.77 (0.74-0.80) | 0.72 (0.68-0.75) |  |
|  | 6 mo | 336 | 0.84 (0.78-1.00) | 209 | 0.84 (0.78-1.00) | 127 | 0.84 (0.78-1.00) | 0.886 | 0.82 (0.79-0.85) | 0.84 (0.81-0.88) |  |
|  | 12 mo | 342 | 1.00 (0.81-1.00) | 215 | 1.00 (0.81-1.00) | 127 | 1.00 (0.81-1.00) | 0.984 | 0.85 (0.82-0.88) | 0.87 (0.83-0.90) |  |
| EQ-5D VAS | 2 we | 359 | 70 (60-80) | 226 | 75 (65-80) | 133 | 70 (60-80) | 0.082 | 72 (69-74) | 70 (68-73) |  |
|  | 6 we | 367 | 75 (65-80) | 231 | 75 (70-80) | 136 | 70 (60-80) | **0.003** | 74 (72-77) | 72 (69-74) |  |
|  | 3 mo | 348 | 80 (70-85) | 219 | 80 (70-85) | 129 | 75 (70-83) | 0.053 | 76 (74-78) | 74 (72-77) |  |
|  | 6 mo | 337 | 80 (70-90) | 209 | 80 (75-90) | 128 | 80 (70-90) | 0.920 | 78 (76-80) | 79 (76-82) |  |
|  | 12 mo | 344 | 80 (75-90) | 217 | 80 (75-90) | 127 | 80 (70-90) | 0.174 | 80 (78-82) | 79 (76-82) |  |
| Shoulder abduction (º) | 2 we | 218 | 40 (10-74) | 161 | 55 (30-80) | 57 | 0 (0-30) | **<0.001** | **47 (41-53)** | **14 (5-22)** |  |
|  | 6 we | 361 | 60 (40-100) | 232 | 80 (50-125) | 129 | 40 (19-55) | **<0.001** | **84 (79-89)** | **41 (35-48)** |  |
|  | 3 mo | 350 | 90 (60-135) | 220 | 105 (73-145) | 130 | 70 (45-95) | **<0.001** | **105 (99-110)** | **77 (70-84)** |  |
|  | 6 mo | 334 | 130 (95-155) | 207 | 140 (105-160) | 127 | 125 (90-150) | **0.002** | 123 (118-129) | 118 (112-125) |  |
|  | 12 mo | 341 | 150 (115-160) | 215 | 150 (115-165) | 126 | 145 (114-155) | 0.099 | 133 (128-139) | 136 (129-141) |  |
| Shoulder anteflexion (º) | 2 we | 213 | 40 (5-75) | 158 | 53 (25-85) | 55 | 0 (0-15) | **<0.001** | **48 (42-53)** | **7 (-1-16)** |  |
|  | 6 we | 360 | 70 (30-114) | 232 | 90 (56-130) | 128 | 28 (10-55) | **<0.001** | **89 (84-94)** | **37 (30-43)** |  |
|  | 3 mo | 350 | 105 (70-135) | 220 | 118 (90-145) | 130 | 80 (47-120) | **<0.001** | **111 (106-116)** | **81 (75-88)** |  |
|  | 6 mo | 334 | 135 (109-155) | 207 | 140 (110-155) | 127 | 125 (100-145) | **0.001** | 128 (122-133) | 123 (116-130) |  |
|  | 12 mo | 341 | 145 (125-160) | 215 | 150 (125-160) | 126 | 140 (120-155) | 0.060 | 135 (130-140) | 136 (130-143) |  |
| Shoulder external rotation (º) | 2 we | 210 | 25 (0-53) | 156 | 40 (15-60) | 54 | -30 (-50-0) | **<0.001** | **35 (32-39)** | **-21 (-27- -15)** |  |
|  | 6 we | 356 | 45 (20-65) | 231 | 60 (40-70) | 125 | 20 (0-38) | **<0.001** | **53 (49-56)** | **17 (13-22)** |  |
|  | 3 mo | 347 | 52 (40-70) | 220 | 60 (45-75) | 127 | 45 (25-60) | **<0.001** | **58 (54-61)** | **41 (36-45)** |  |
|  | 6 mo | 334 | 60 (50-75) | 207 | 65 (55-80) | 127 | 60 (45-70) | **<0.001** | 64 (60-67) | 57 (53-61) |  |
|  | 12 mo | 341 | 65 (55-79) | 215 | 70 (55-80) | 126 | 65 (50-75) | **0.016** | 66 (63-69) | 62 (58-67) |  |
| Shoulder internal rotation (º) | 2 we | 204 | 53 (30-70) | 149 | 60 (38-70) | 55 | 50 (25-60) | 0.264 | 47 (44-50) | 46 (41-51) |  |
|  | 6 we | 350 | 60 (45-70) | 228 | 60 (45-70) | 122 | 60 (40-65) | 0.214 | 54 (51-57) | 53 (49-57) |  |
|  | 3 mo | 344 | 60 (45-70) | 218 | 60 (45-70) | 126 | 60 (45-70) | 0.114 | 58 (55-60) | 57 (53-60) |  |
|  | 6 mo | 333 | 65 (50-70) | 207 | 65 (55-75) | 126 | 60 (50-70) | **0.007** | 63 (60-66) | 60 (57-64) |  |
|  | 12 mo | 340 | 65 (50-75) | 215 | 65 (50-75) | 125 | 65 (50-75) | 0.592 | 63 (60-66) | 64 (61-68) |  |
| Elbow flexion (º) | 2 we | 204 | 105 (90-124) | 153 | 110 (90-125) | 51 | 90 (90-110) | **<0.001** | **108 (106-111)** | **95 (91-99)** |  |
|  | 6 we | 359 | 130 (120-140) | 230 | 135 (125-140) | 129 | 120 (100-130) | **<0.001** | **131 (129-134)** | **114 (112-117)** |  |
|  | 3 mo | 348 | 135 (130-140) | 220 | 140 (133-141) | 128 | 135 (120-140) | **<0.001** | **137 (135-139)** | **129 (126-132)** |  |
|  | 6 mo | 334 | 140 (135-145) | 207 | 140 (135-145) | 127 | 140 (130-145) | 0.112 | 139 (136-141) | 137 (134-140) |  |
|  | 12 mo | 341 | 140 (135-145) | 215 | 140 (135-145) | 126 | 140 (135-145) | 1.000 | 140 (137-142) | 139 (136-141) |  |
| Elbow extension (º) | 2 we | 204 | -30 (-45- -15) | 153 | -25 (-40- -15) | 51 | -50 (-75- -30) | **<0.001** | -30 (-33- -28) | -50 (-54- -46) |  |
|  | 6 we | 359 | -10 (-30- -5) | 230 | -6 (-20-0) | 129 | -25 (-45- -15) | **<0.001** | -13 (-15- -11) | -30 (-33- -27) |  |
|  | 3 mo | 349 | -5 (-15-0) | 220 | 0 (-10-5) | 129 | -10 (-18-0) | **<0.001** | -5 (-7- -3) | -11 (-14- -8) |  |
|  | 6 mo | 334 | 0 (-5-5) | 207 | 0 (-5-5) | 127 | 0 (-5-0) | **0.001** | -2 (-5-0) | -3 (-6-0) |  |
|  | 12 mo | 341 | 0 (0-5) | 215 | 0 (0-5) | 126 | 0 (-5-5) | **0.009** | 0 (-2-3) | -1 (-4-2) |  |
| Elbow pronation (º) | 2 we | 222 | 80 (75-85) | 162 | 83 (75-85) | 60 | 75 (60-80) | **<0.001** | **78 (76-80)** | **63 (60-65)** |  |
|  | 6 we | 364 | 85 (80-85) | 232 | 85 (80-85) | 132 | 80 (75-85) | **<0.001** | **82 (81-84)** | **77 (75-79)** |  |
|  | 3 mo | 351 | 85 (80-90) | 221 | 85 (80-90) | 130 | 80 (80-85) | **<0.001** | 84 (82-85) | 81 (79-83) |  |
|  | 6 mo | 334 | 85 (80-90) | 207 | 85 (80-90) | 127 | 85 (80-85) | 0.052 | 83 (82-85) | 83 (81-85) |  |
|  | 12 mo | 341 | 85 (80-90) | 215 | 85 (84-90) | 126 | 85 (80-86) | **0.004** | 85 (83-86) | 83 (81-85) |  |
| Elbow supination (º) | 2 we | 222 | 75 (60-85) | 162 | 77 (69-85) | 60 | 60 (33-75) | **<0.001** | **71 (68-73)** | **52 (48-55)** |  |
|  | 6 we | 364 | 80 (71-85) | 232 | 85 (75-87) | 132 | 75 (62-85) | **<0.001** | **79 (77-81)** | **71 (69-74)** |  |
|  | 3 mo | 351 | 85 (80-85) | 221 | 85 (80-90) | 130 | 80 (75-85) | **<0.001** | 82 (80-84) | 78 (76-81) |  |
|  | 6 mo | 334 | 85 (80-90) | 207 | 85 (80-90) | 127 | 85 (80-90) | 0.083 | 82 (80-84) | 81 (79-84) |  |
|  | 12 mo | 341 | 85 (80-90) | 215 | 85 (80-90) | 126 | 85 (80-89) | **0.025** | 83 (81-85) | 83 (80-86) |  |

Data for the univariate analysis are presented as median (P_25_-P_75_), data for the repeated measure multivariable analysis are shown as estimated marginal mean with 95% confidence interval.

N* represents the number of patients for whom data were available per follow-up moment.

DASH, Disabilities of the Arm, Shoulder, and Hand; EQ-5D, EuroQoL-5D; MCS, Mental Component Summary; Mo, month; NRS, Numerical Rating Scale; PCS, Physical Component Summary; SF-36, Short Form-36; US, Utility Score; VAS, Visual Analog Scale; We, week.
